# Photochemical Formation and Electronic Structure of an Alkane σ-Complex from Time-Resolved Optical and X-ray Absorption Spectroscopy

**DOI:** 10.1021/jacs.4c02077

**Published:** 2024-05-07

**Authors:** Raphael M. Jay, Michael R. Coates, Huan Zhao, Marc-Oliver Winghart, Peng Han, Ru-Pan Wang, Jessica Harich, Ambar Banerjee, Hampus Wikmark, Mattis Fondell, Erik T. J. Nibbering, Michael Odelius, Nils Huse, Philippe Wernet

**Affiliations:** † Department of Physics and Astronomy, Uppsala University, 75120 Uppsala, Sweden; ‡ Department of Physics, AlbaNova University Center, Stockholm University, 10691 Stockholm, Sweden; § Center for Free-Electron Laser Science, Department of Physics, University of Hamburg, 22761 Hamburg, Germany; ∥ Max Born Institute for Nonlinear Optics and Short Pulse Spectroscopy, 12489 Berlin, Germany; ⊥ Institute for Methods and Instrumentation for Synchrotron Radiation Research, Helmholtz-Zentrum Berlin für Materialien und Energie GmbH, 12489 Berlin, Germany

## Abstract

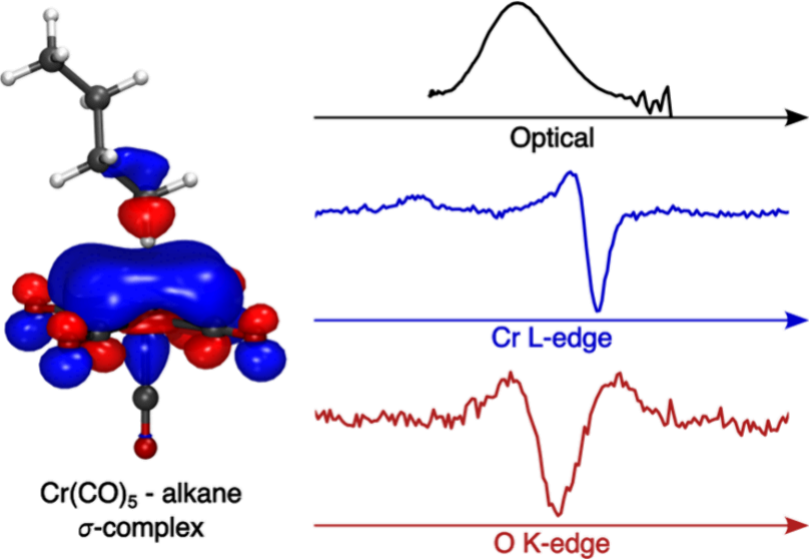

C–H bond activation reactions with transition metals typically proceed via the formation of alkane σ-complexes, where an alkane C–H σ-bond binds to the metal. Due to the weak nature of metal–alkane bonds, σ-complexes are challenging to characterize experimentally. Here, we establish the complete pathways of photochemical formation of the model σ-complex Cr(CO)_5_-alkane from Cr(CO)_6_ in octane solution and characterize the nature of its metal–ligand bonding interactions. Using femtosecond optical absorption spectroscopy, we find photoinduced CO dissociation from Cr(CO)_6_ to occur within the 100 fs time resolution of the experiment. Rapid geminate recombination by a fraction of molecules is found to occur with a time constant of 150 fs. The formation of bare Cr(CO)_5_ in its singlet ground state is followed by complexation of an octane molecule from solution with a time constant of 8.2 ps. Picosecond X-ray absorption spectroscopy at the Cr L-edge and O K-edge provides unique information on the electronic structure of the Cr(CO)_5_-alkane σ-complex from both the metal and ligand perspectives. Based on clear experimental observables, we find substantial destabilization of the lowest unoccupied molecular orbital upon coordination of the C–H bond to the undercoordinated Cr center in the Cr(CO)_5_-alkane σ-complex, and we define this as a general, orbital-based descriptor of the metal–alkane bond. Our study demonstrates the value of combining optical and X-ray spectroscopic methods as complementary tools to study the stability and reactivity of alkane σ-complexes in their role as the decisive intermediates in C–H bond activation reactions.

## Introduction

Alkane σ-complexes play a critical role in C–H bond activation reactions with transition metal complexes. They constitute the decisive intermediates in which an alkane C–H bond directly coordinates and interacts with the transition metal.^1−3^ Due to their low polarizability, however, alkanes are generally poor electron donors and acceptors. The bond strength between the metal and the alkane C–H bond in σ-complexes is therefore typically very weak. Still, the subtle degree of polarization induced in the C–H bond through binding to the metal site in σ-complex intermediates critically determines the reactivity toward bond cleavage in C–H activation reactions.^4^ In pursuit of understanding this unusual bonding configuration, a rich literature of complexes, in which alkane C–H bonds act as ligands bound to a metal center, has emerged over the decades.^3,5−7^

**Scheme 1:**
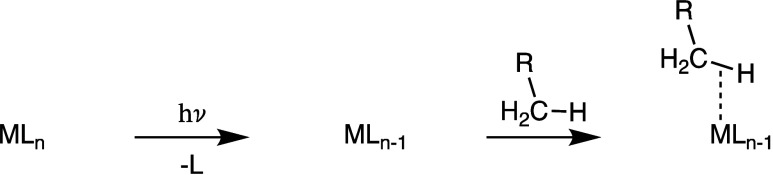
Reaction Scheme of Photochemical Alkane σ-Complex Formation

Various routes exist toward preparation of alkane σ-complexes. Direct preparation in the solid state has been achieved by hydrogenation of metal-bound alkenes^8,9^ as well as in solution via protonation of a metal-bound alkyl group at very low temperature.^10^ The most common routes to preparing alkane σ-complexes, however, are photochemical ones^3^ (see Scheme 1). The photoinduced elimination of a ligand from certain transition metal complexes leads to the creation of an undercoordinated and highly reactive species capable of binding an alkane from solution. This type of photochemical preparation has been the basis for a wide range of spectroscopic investigations of alkane σ-complexes. Early flash photolysis experiments in low-temperature matrices as well as solution unambiguously established that alkanes can indeed act as ligands in a metal complex.^11−14^ Later, nuclear magnetic resonance (NMR) and time-resolved infrared (IR) spectroscopy, in particular, were instrumental in characterizing the structure of alkane σ-complexes as well as their mechanistic role in C–H activation reactions.^15−22^

As the precursor to Cr(CO)_5_-alkane, one of the first ever observed alkane σ-complexes,^11,13,14^ chromium hexacarbonyl (Cr(CO)_6_), has long served as a model systems for both developing a general mechanistic understanding of photoinduced ligand exchange and gaining specific insight into the nature and properties of alkane σ-complexes.^6^ However, while constituting a thoroughly studied model system, experimentally resolving the ultrafast time scales of the formation of Cr(CO)_5_-alkane σ-complexes has proven difficult. NMR spectroscopy is intrinsically slow compared to ligand-exchange dynamics and can only probe species that are metastable on nano- to millisecond time scales. Time-resolved IR spectroscopy accesses the time scales necessary to detect σ-complexes but has limited sensitivity to their electronic structure and to the sub-picosecond regime of excited-state and dissociation dynamics due to the broad and overlapping IR absorption bands of vibrationally hot species on these time scales.

Here, we combine femtosecond optical absorption and picosecond X-ray absorption spectroscopy^23,24^ to determine, based on the complementary time scales and properties these methods access, the photochemical pathway of formation and the bonding characteristics of σ-complexes with Cr(CO)_6_ in alkane solution. Femtosecond optical absorption spectroscopy has been used before to follow the initial steps in the ligand-exchange dynamics of Cr(CO)_6_ and other transition metal complexes in various solvents including alkanes.^25−32^ Due to insufficient time resolution and detection sensitivity, however, these investigations were unable to robustly resolve and assign the intermediates along the alkane σ-complex formation from Cr(CO)_6_ observed on the femtosecond time scale.^25,30^ To date, the pathway of photochemical formation of Cr(CO)_5_-alkane and many other σ-complexes has not been fully resolved. For instance, it has remained unclear what the time scale of CO dissociation in solution is, whether CO dissociation produces a bare Cr(CO)_5_ fragment in solution before binding to an alkane, and, if so, whether Cr(CO)_5_ is present in its ground or an excited state. Answering these questions is critical for developing a fundamental mechanistic understanding of ligand-exchange reactions as the basis for alkane σ-complex formation in photochemical C–H activation reactions with transition metals.

Time-resolved X-ray absorption spectroscopy has previously been shown to provide information highly complementary to the information provided by time-resolved optical and infrared absorption spectroscopy. Measuring the extended absorption fine structure (EXAFS) at the tungsten L-edge has recently allowed Bartlett et al. to determine metal–alkane bond lengths in W(CO)_5_-alkane σ-complexes.^33^ By evaluating the character of main-edge transitions in the rhodium L-edge absorption spectrum, the metal–alkane orbital interactions upon formation of rhodium–alkane σ-complexes as well as the ensuing C–H bond cleavage via oxidative addition have recently been observed.^34^ The direct access of X-ray absorption spectroscopy to the relevant metal d-derived orbitals by the 2p → nd transitions underlying transition metal L-edges allows evaluating the degree to which metal d orbitals hybridize with incoming alkane C–H bonds. Such properties of alkane σ-complexes, which have thus far mostly been studied using theory,^35−38^ can now be validated experimentally.^34^ Here, we use the respective capabilities of femtosecond optical absorption and picosecond X-ray absorption spectroscopy to unambiguously establish the full photochemical pathways of Cr(CO)_6_ dissociation in octane solution and to characterize the electronic structure of the resulting Cr(CO)_5_-alkane σ-complex within novel orbital-based descriptors.

## Methods

### Materials

Cr(CO)_6_ was prepared in *n*-octane (purchased from Sigma-Aldrich) at concentrations of 7 mM for the time-resolved transient optical and 20 mM for the X-ray absorption measurements. To achieve the concentration of the X-ray absorption measurements, the sample was stirred and put into a sonication bath for up to ∼1 h.

### Transient UV–Visible Absorption Spectroscopy

Time-resolved UV–visible measurements were conducted using a 3 kHz Ti:sapphire amplified laser system (Spectra Physics Spitfire Ace, 90 fs, 800 nm). The sample was prepared as a free-flowing 300 μm thick wire-guided liquid sheet. The sample was optically excited by the third harmonic of the fundamental laser wavelength with a fluence of 4.6 mJ/cm^2^. A supercontinuum pulse (generated in a 1 mm calcium fluoride plate) was used to probe the absorbance change in transmission using a prism-based imaging spectrograph and a CCD detector, while a second supercontinuum pulse served as a reference. The data were chirp-corrected by using the delay at half-rise of the signal onset for every wavelength. The time resolution as determined by the rise time of the transient absorption signals was ∼100 fs. Pump–probe measurements of the pure octane solvent under the same experimental conditions showed a negligible contribution of a coherent artifact to the pump-induced signal around zero delay.

### Time-Resolved X-ray Absorption Spectroscopy

The time-resolved X-ray absorption measurements were performed at the UE52-SGM beamline^39^ of the BESSY II synchrotron. The X-ray absorption data were acquired in transmission geometry using a flatjet sample delivery system.^40,41^ The liquid sample was transported into the experimental vacuum chamber via two colliding jets, thereby forming a thin liquid sheet. To form a stable octane sheet, flow rates were kept between 4.5 and 5 mL/min using a pair of nozzles with a diameter of ∼50 μm. The thickness of the liquid sheet was between 4.3 and 4.6 μm throughout all measurements as estimated from the comparison of the transmissions at 528 eV (O K-edge) and 573 eV (Cr L-edge) to tabulated values.^42^ The bandwidth of the incidence X-rays was ∼200 meV at the O K-edge and ∼250 meV at the Cr L-edge. The sample was optically excited by the fourth harmonic of a fiber laser (Amplitude Tangerine, 1030 nm, 350 fs) at 258 nm wavelength. The repetition rate of the laser was set to 208 kHz for all measurements. The laser pulse energy was ∼5 μJ at a spot size of ∼60 × 100 μm^2^ at the sample, amounting to an overall laser fluence of ∼100 mJ/cm^2^. The time resolution as derived from the rise time of the transient X-ray signals was ∼45 ps (determined by the X-ray pulse duration).

### Computational Details

Geometry optimizations were performed with the Gaussian 16 quantum chemistry suite^43^ on the level of density functional theory (DFT) using the TPSSh functional^44,45^ and the def2-TZVP^46,47^ basis set. Cr(CO)_6_ was optimized in *O*_*h*_ symmetry, Cr(CO)_5_ in both *C*_4*v*_ and *C*_2*v*_ symmetry, and Cr(CO)_5_-butane in *C*_1_ symmetry. The butane-based σ-complex was used instead of the octane-based σ-complex to reduce the computational cost. Solvent effects were incorporated in the geometry optimizations by implicit solvation given by the conductor-like polarizable continuum model (CPCM)^48,49^ using a dielectric constant corresponding to octane (ε = 1.9406). Based on these structures, the Cr L-edge spectra were simulated using the restricted active space self-consistent field^50^ (RASSCF) wave function, while the optical and O K-edge spectra were simulated using time-dependent density functional theory (TD-DFT). The details of each method are described below.

The RASSCF Cr L-edge absorption spectra were simulated in OpenMolcas.^51^ For all spectra, the active space was constructed by rotating the Cr 2p orbitals (frozen at the Hartree–Fock level) into the RAS1 subspace and allowing at most 1 hole in the RAS1 subspace, rotating occupied valence orbitals in RAS2 and low-lying virtual orbitals in RAS3, allowing for at most 2 electrons in the RAS3 subspace. Starting with Cr(CO)_6_, 16 electrons are distributed in 19 orbitals corresponding to the 1t_1u_ (2p) core orbitals, 5e_g_ (σ CO-d_*x*^2^–*y*^2^_ and σ CO-d_*z*^2^_) and 2t_2g_ (d-π CO*) occupied orbitals, and the 9t_1u_ (π CO*), 2t_2u_ (π CO*), 3t_2g_ (π CO*-d), and 6e_g_ (d_*x*^2^–*y*^2^_-σ CO and d_*z*^2^_-σ CO) unoccupied orbitals, resulting in a wave function denoted RAS(16,1,2;3,5,11). For the RASSCF L-edge spectra, zeroth-order relativistic effects were incorporated by the Douglas–Kroll–Hess Hamiltonian^52^ for the ANO-RCC-VTZP basis set^53,54^ used for all atoms. Eighty singlet and 80 triplet valence states were independently solved for by a state-averaged RASSCF wave function. A total of 240 singlet and 480 triplet core-excited states were independently solved for by a state-averaged RASSCF wave function by using the HEXS keyword in the RASSCF module in OpenMolcas. The subsequent spin–orbit coupled L-edge spectra were obtained by the RASSI module.^55^ All other complexes (Cr(CO)_5_ (*C*_4*v*_), Cr(CO)_5_ (*C*_2*v*_), and Cr(CO)_5_-butane) employed the same active space allowing for changes in orbital character. All spectrum calculations were performed in *C*_1_ symmetry.

TD-DFT calculations of the optical and O K-edge absorption spectra were performed using the ORCA quantum chemistry package^56,57^ at the B3LYP level of theory^58,59^ with the def2-TZVP basis set.^46,47^ For computational efficiency, the RIJCOSX approximation^60^ was used. For the optical absorption spectra, 30 roots were calculated. For the O K-edge absorption spectra, the Pipek–Mezey orbital localization scheme^61^ was used, and the excitation window was restricted to only include the O 1s orbitals. The number of calculated roots was set to 30 roots per O atom for the different species.

The simulated optical absorption spectra were generated by convolving the calculated transitions with a Gaussian function with a width of 0.3 eV full width at half-maximum (fwhm) to account for the experimental and inhomogeneous broadening. The simulated X-ray absorption spectra were generated by convolving the calculated transitions with a pseudo-Voigt function. The Lorentz contribution to the Voigt function accounts for the tabulated lifetime broadening^62^ of 0.32 eV fwhm at the Cr L_3_-edge, 0.76 eV at the Cr L_2_-edge, and 0.18 eV at the O K-edge. The Gaussian contribution of 0.4 eV fwhm throughout the Cr L-edge and 0.8 eV at the O K-edge accounts for the experimental and inhomogeneous broadening. To align the calculated spectra with the experimental spectra, a uniform shift of −6.47 eV was applied at the Cr L-edge, whereas a shift of +13.7 eV was applied at the O K-edge. This is done to compensate for errors accumulated from the approximations used in simulating the X-ray absorption spectra. The relative shifts between each species are therefore preserved within each method used.

## Results and Discussion

### Transient UV–Visible Absorption Spectroscopy

**Figure 1:**
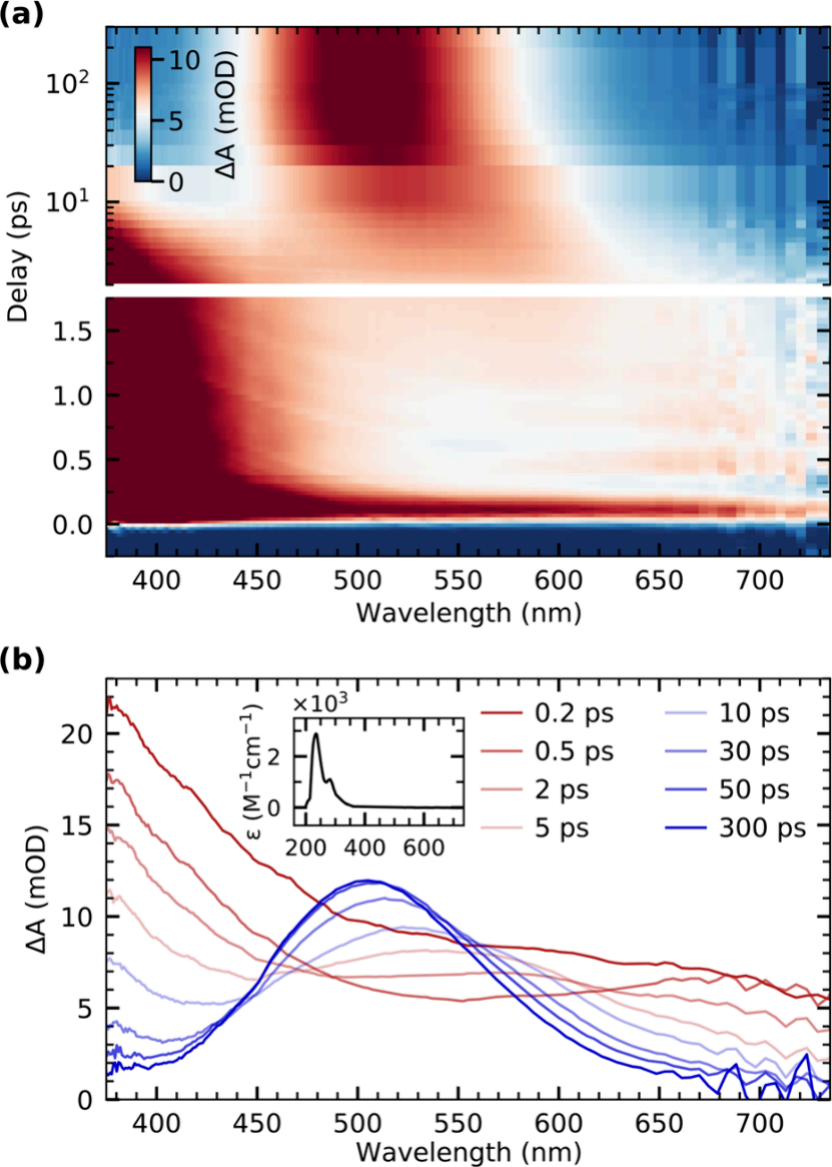
(a) Overview of the transient optical absorption data of Cr(CO)_6_ in octane solution following photoexcitation with a 266 nm laser pulse. (b) Transient absorption spectra for selected pump–probe delays displaying an initial broad absorption feature throughout the observed spectral range followed by formation of a transient absorption band centered at around 500 nm. The steady-state absorption spectrum of Cr(CO)_6_ is shown in the inset.

Figure 1a shows an overview of the transient optical absorption data of Cr(CO)_6_ in octane solution covering pump–probe delays up to 300 ps. Representative cuts along the spectral range are displayed in Figure 1b for selected pump–probe delays. The inset in Figure 1b shows negligible steady-state absorption of Cr(CO)_6_ in the spectral range probed in the transient absorption measurements. The time-resolved data qualitatively reproduce earlier transient absorption data of Cr(CO)_6_ and other metal hexacarbonyl complexes in various solvents including alkanes.^25,29,30^ With improved detection sensitivity and temporal resolution, our measurements now reveal new information. Within the time resolution of the experiment, a broad absorption appears, which stretches across the observed spectral range at the earliest measured time delays and which decreases in spectral bandwidth within the first few hundred femtoseconds. At the same time, a strong absorption band can be observed for wavelengths shorter than 450 nm (Figure 1a). Additionally, oscillatory signals are apparent at delay times below 2 ps in the spectral range between 600 and 720 nm (oscillatory signals are faintly visible below 450 nm as well, as detailed below). Within a few picoseconds, we see a broad transient absorption band emerging in the red part of the spectrum and gradually moving to shorter wavelengths concomitant with the decay of the absorption below 450 nm (Figure 1a). An additional blue-shift as well as a narrowing of the absorption band around 500 nm takes place on the time scale of tens to hundreds of picoseconds (Figure 1b). This prominent absorption band was previously observed and identified as a transition between the highest occupied molecular orbital (HOMO) and the lowest unoccupied molecular orbital (LUMO) of the solvent-complexed Cr(CO)_5_ fragment in its electronic ground state.^13^ Notably, the energy of this transition was shown to scale with the bond strength toward the associated molecule,^11,14^ and we will use this property later in our analysis.

**Figure 2:**
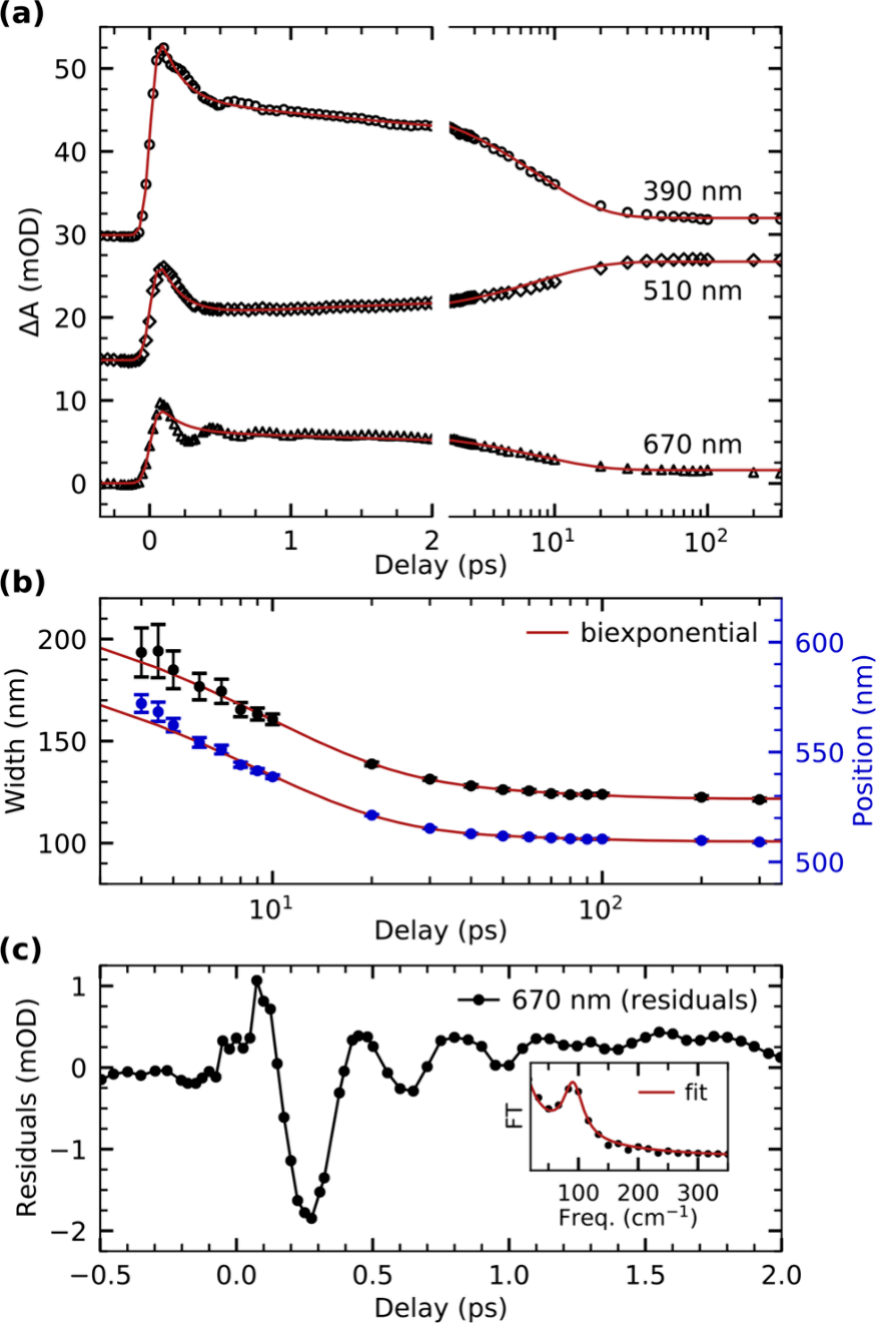
(a) Delay traces extracted at three different wavelengths and modeled with a global fit based on a biexponential model. (b) Width and position of the transient absorption band arising after photoexcitation in the range of 480 to 640 nm as a function of pump–probe delay. The evolution of both width and position is modeled by a biexponential function. (c) Residuals of the global fit of the delay trace at 670 nm from panel (a) with a Fourier transfer (FT) analysis of the residuals depicted in the inset. The fit is the sum of a Lorentzian line shape and, to account for the background, the inverse frequency ν, *a*/ν, where *a* is a fit parameter.

Cuts along the pump–probe delay axis for selected wavelengths are displayed in Figure 2a for closer inspection. The three delay traces are modeled by a global fit based on a biexponential model, yielding a fast time constant of 150 ± 10 fs and a slower time constant of 7.7 ± 0.3 ps. The first time constant of 150 fs, which describes the initial decrease of the broad absorption across the observed spectral range, quantitatively reproduces dynamics reported in earlier femtosecond IR measurements,^63^ where the recovery of the bleach signal on the same time scale was assigned to geminate recombination of dissociated CO with the Cr(CO)_5_ fragment. The second time constant of 7.7 ps we instead assign to the association of an octane molecule from solution in agreement with previous femtosecond studies on Cr(CO)_6_ as well as other metal carbonyls.^29,30,32,34^

The results of the analysis of the width and position of this HOMO → LUMO absorption band are shown in Figure 2b (see Supporting Information for details of the analysis). The evolution of both width and position is well described by a biexponential temporal behavior, where the fast component of 8.6 ± 0.6 ps is in good agreement with the time scale of 7.7 ± 0.3 ps determined from the global fit of the delay traces in Figure 2a and assigned to σ-complex formation. Given that the two values stem from independent observables, we define the mean of 8.2 ± 0.4 ps as the time constant for σ-complex formation. The slower time constant of 53 ± 12 ps of the biexponential fit is assigned to thermalization of the σ-complex with the solvent environment, in agreement with previous measurements of other metal carbonyls in solution.^29,30,64^ We find the final peak position of the Cr(CO)_5_-alkane σ-complex after ∼100 ps to be at 510 nm. This wavelength is in excellent agreement with the band position of Cr(CO)_5_ bound to an alkane in solution and in noble gas matrices as derived from flash photolysis measurements.^12−14^

**Scheme 2:**
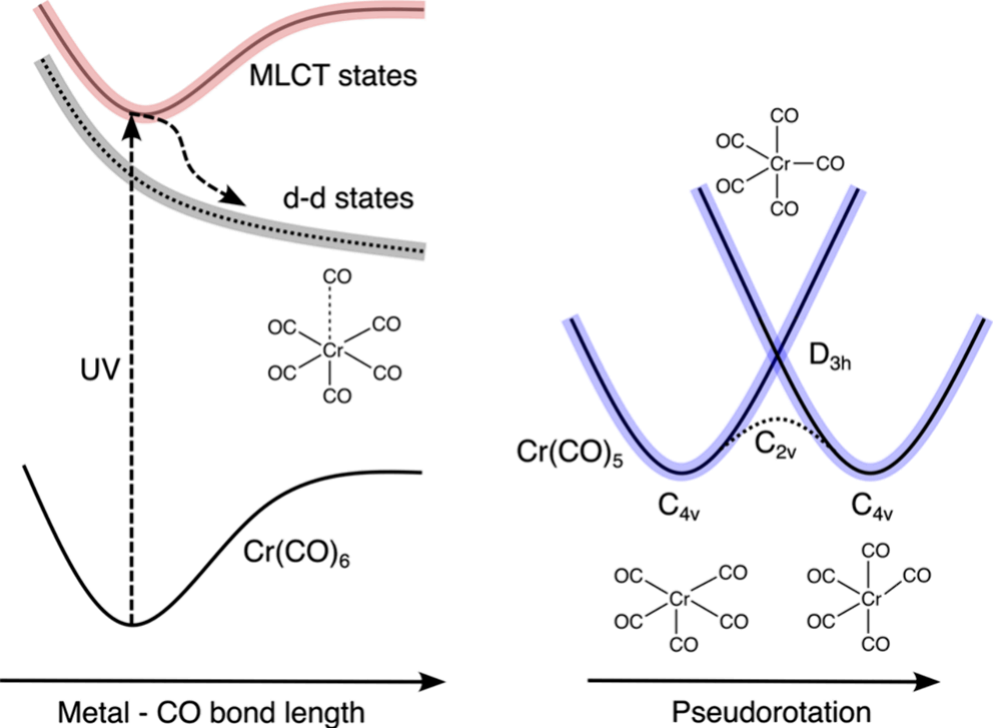
Schematic Depiction of the Potential Energy Surfaces along Photoinduced CO Dissociation from Cr(CO)_6_ and Subsequent Formation of the Cr(CO)_5_ Fragment (Adapted from Ref (68))

The oscillatory signal observed right after the UV excitation in the red part of the overview map in Figure 1a can be isolated by evaluating the residuals from the fit of the delay trace at 670 nm displayed in Figure 2c. A Fourier analysis of these data (shown in the inset) reveals a single frequency of 91 ± 1 cm^–1^. This is in good agreement with the coherent oscillations observed in the photochemistry of gas-phase Cr(CO)_6_.^65−67^ In the gas phase, these oscillations have been assigned to equatorial Cr-CO bending modes in the S_0_ ground state of Cr(CO)_5_ with *C*_4*v*_ symmetry. These modes are thought to be populated following the passing of the S_1_ excited state of Cr(CO)_5_, which exhibits *D*_3*h*_ symmetry^65−68^ (see Scheme 2). We measure a dephasing time of 830 ± 50 fs for the coherent oscillations observed here, in excellent agreement with the value of 815 ± 20 fs measured in the gas phase.^65^ The matching oscillation frequencies and dephasing times clearly indicate that the same equatorial Cr-CO bending modes of Cr(CO)_5_ are populated in solution and in the gas phase. In solution, and in cases where Cr(CO)_5_ does not undergo geminate recombination, our findings thus suggest that the octane solvent has no measurable influence on the nature of the initial wavepacket dynamics along the dissociation pathway, nor does the solvent seem to influence the dephasing time of the oscillations. As in the gas phase, we think, the excess vibrational energy is first dissipated via intramolecular vibrational energy redistribution (IVR) to lower-energy modes of the solute. However, the solvent must already provide a bath for energy dissipation within the first picoseconds because, unlike in the gas phase,^65,67^ additional ligand dissociation is not observed in solution. It is thus conceivable that the time constant of 8.2 ps, which we assign to the formation of the Cr(CO)_5_-alkane σ-complex, includes a component that corresponds to a further thermalization of the bare Cr(CO)_5_ with the solvent environment and that thermalization and alkane association are concerted processes.

It is noteworthy that the coherent oscillations we observe appear in the far-red part of the transient absorption spectrum. Previous flash photolysis experiments of Cr(CO)_5_ in a neon matrix^13^ as well as in perfluorocyclohexane solution^14^ observed the fingerprint HOMO → LUMO absorption band in the range of 620 to 630 nm. Because neon and perfluorocyclohexane have been considered to be noninteracting moieties, this has been interpreted to be the signature of bare Cr(CO)_5_. The absorption spectrum at 0.5 ps in Figure 1b exhibits an absorption band at even higher wavelengths at around 670 nm, which indicates that even neon and perfluorocyclohexane weakly bind the undercoordinated metal center and shift the HOMO → LUMO absorption band to higher energy with respect to the band position measured here. Besides this sensitivity to the nature of the moiety bound to the metal center, it has further previously been shown that the energy of the HOMO → LUMO absorption band also depends on structural distortions and the specific symmetry of the Cr(CO)_5_ fragment.^13,69,70^ Both our observations of a transient absorption band in the far-red absorption range and the modulation of the band by the same characteristic oscillations as in the gas phase therefore strongly point to the formation of bare Cr(CO)_5_ in solution.

**Figure 3:**
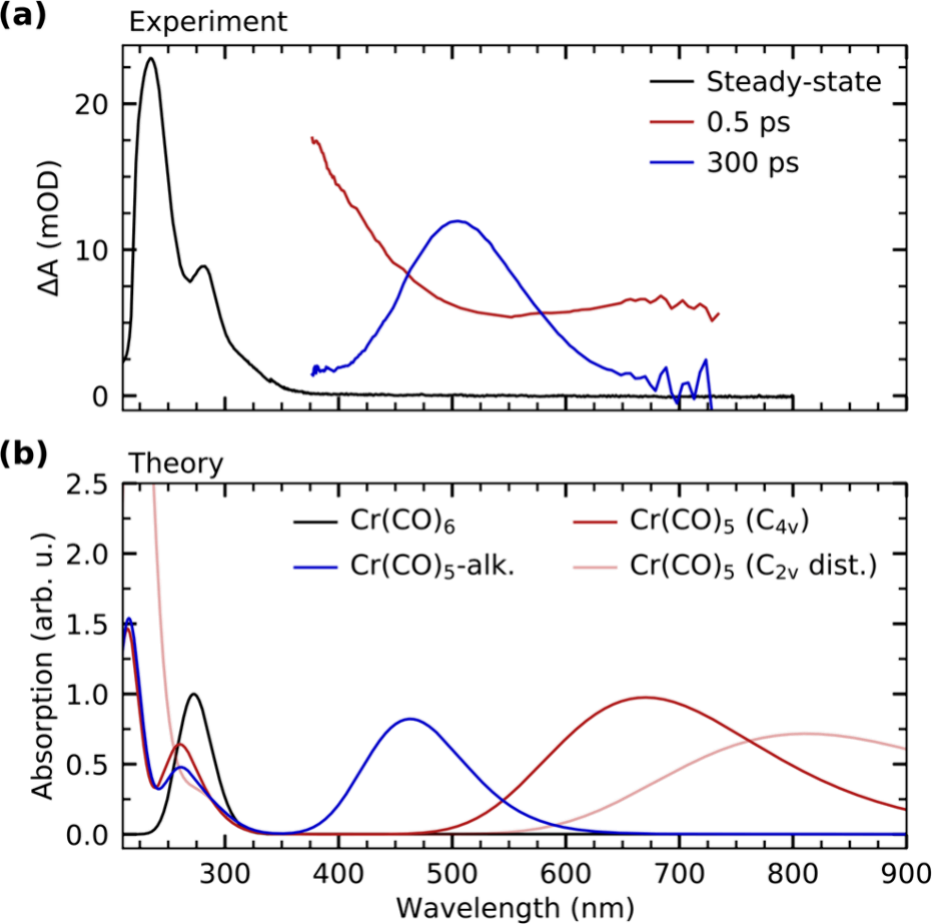
(a) Steady-state optical absorption spectrum of Cr(CO)_6_ compared with transient optical absorption spectra at 0.5 and 300 ps pump–probe delay. For comparison, the steady-state spectrum is scaled. (b) Calculated absorption spectra of Cr(CO)_6_, Cr(CO)_5_ in *C*_4*v*_ and a *C*_2*v*_ distorted symmetry, and the Cr(CO)_5_-alkane σ-complex at the TD-DFT level of theory. The calculated spectrum of Cr(CO)_6_ is scaled so that the transition at ∼290 nm is 1. All other species are scaled accordingly.

As we show in the following, the purely experimental assignments of the transient optical absorption bands to specific species are confirmed by calculated spectra. Figure 3a contains the experimental steady-state absorption spectrum of Cr(CO)_6_ as well as transient absorption spectra of bare Cr(CO)_5_ (delay of 0.5 ps) and the Cr(CO)_5_-alkane σ-complex (delay of 300 ps). Our simulated spectra of Cr(CO)_6_, Cr(CO)_5_, and the Cr(CO)_5_-octane σ-complex calculated at the TD-DFT level of theory are shown in Figure 3b. Confirming our experimental assignments, the long-lived transient band at 510 nm can be identified with theory as the fingerprint of the Cr(CO)_5_-alkane σ-complex. Bare Cr(CO)_5_ in its *C*_4*v*_ ground-state geometry instead exhibits an absorption band centered at ∼670 nm. Our calculations also indicate a strong sensitivity of the position of the Cr(CO)_5_ HOMO → LUMO absorption band to structural distortions. A shift from 670 to 800 nm is calculated when going from the *C*_4*v*_ ground-state geometry of Cr(CO)_5_ to a *C*_2*v*_ distorted structure. This distorted structure can be thought to represent a prevalent structure of vibrationally excited Cr(CO)_5_ where Cr–CO bonds are bent toward the *C*_2*v*_ geometry of the transition state along the pseudorotation coordinate^68^ (compare Scheme 2). In our calculations, the structure was generated by a restricted optimization of Cr(CO)_5_ in *C*_2*v*_ symmetry, where two opposing equatorial CO groups are bent out of plane by a fixed angle of 15°. An oscillatory motion in Cr(CO)_5_ between *C*_4*v*_ and *C*_2*v*_ symmetry along the bending mode of the equatorial CO ligands would thus modulate the intensity in the optical absorption spectrum around 670 nm as observed experimentally. This explains the coherent oscillations on sub-picosecond time scales in this spectral region (see Figure 1a).

We note that the strong absorption band observed during the first picoseconds below 450 nm (see Figure 1a) is not reproduced by theory for any of the calculated species. Still, since the band decays with a time constant of 8.2 ps as the final Cr(CO)_5_-alkane product rises, we can tentatively attribute the absorption band below 450 nm to a transition of bare Cr(CO)_5_. During the first picoseconds, bare Cr(CO)_5_ can be expected to be structurally far from thermal equilibrium. The associated vibrational excitations may then allow the nominally symmetry-forbidden absorption below 450 nm to adopt its substantial oscillator strength. This effect, however, is not reproduced by our level of theory, which is based on vibrational and electronic ground states. Our assignment of the absorption band below 450 nm to bare Cr(CO)_5_ is corroborated in addition by assessing the residuals of the delay trace at 390 nm (see Figure S1 in the Supporting Information). The delay trace at that wavelength exhibits the same, albeit less pronounced, characteristic oscillations within the first picosecond as the trace at 670 nm, which we identified as one of the fingerprints of the bare Cr(CO)_5_ intermediate. This assignment of the band below 450 nm is also consistent with its initial intensity decrease with a time constant of 150 fs. The magnitude of the decrease is ∼30%, which is in good agreement with the previously determined yield for a geminate recombination of 34% for Cr(CO)_5_ in alkane solution.^71^

**Scheme 3:**
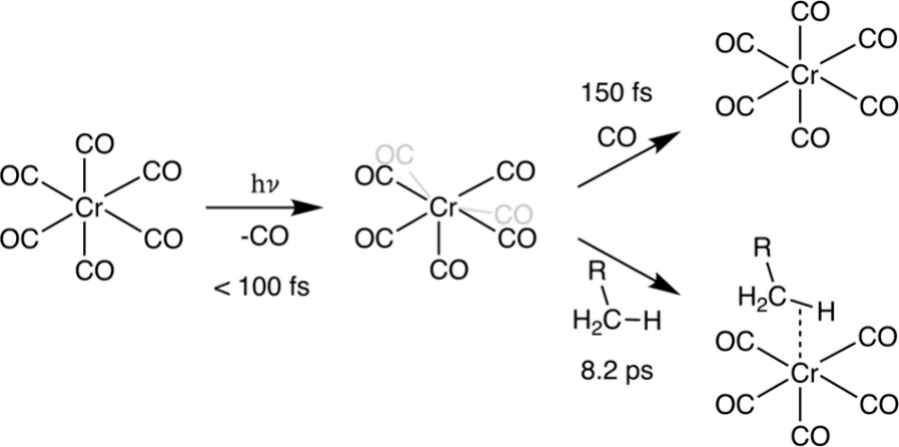
Determined Photochemical Pathway and Associated Time Scales of Alkane σ-Complex Formation Following CO Dissociation from Cr(CO)_6_ in Octane Solution

Our experimental data therefore allow to robustly establish the ultrafast pathway leading to the formation of the Cr(CO)_5_-alkane σ-complex in octane solution (see Scheme 3): As in the gas phase, CO dissociation and formation of Cr(CO)_5_ occur on a time scale below 100 fs^65−67^ and thus within the time resolution of the experiment here. With a time constant of 150 fs, a fraction of bare Cr(CO)_5_ molecules undergoes geminate recombination with CO groups, which have not escaped the first solvation shell following dissociation. Those Cr(CO)_5_ molecules that do not geminately recombine clearly occur in their electronic ground state in *C*_4*v*_ symmetry with characteristic equatorial Cr-CO bending modes, which dephase with a time constant of 830 fs. The Cr(CO)_5_-alkane σ-complex then forms with a time constant of 8.2 ps via a ground-state reaction, in which an octane molecule binds to the bare Cr(CO)_5_. With a time constant of 53 ps, the Cr(CO)_5_-alkane σ-complex vibrationally cools and persists thereafter.

### Time-Resolved X-ray Absorption Spectroscopy

**Figure 4:**
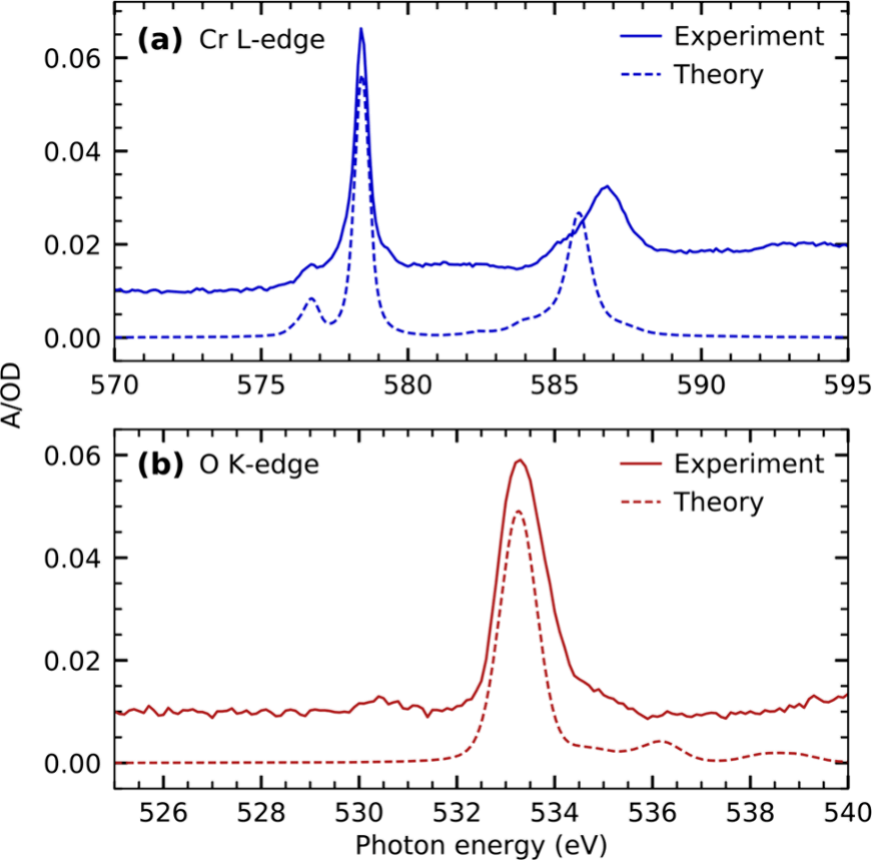
Steady-state X-ray absorption spectra of Cr(CO)_6_ in octane solution measured at the (a) Cr L-edge and (b) O K-edge compared with calculated spectra at the RASSCF and TD-DFT level of theory, respectively.

Figure 4a and b show the steady-state X-ray absorption spectra of Cr(CO)_6_ in octane solution measured at the Cr L-edge and O K-edge, respectively. The shapes of both spectra are in good agreement with previous soft X-ray absorption measurements on Cr(CO)_6_ in 1-pentanol solution.^72^ Below the measurements, spectra calculated at the RASSCF level of theory are shown for the Cr L-edge as well as the TD-DFT level of theory for the O K-edge. The calculations are in good agreement with experiment and can hence be used to assign the observed spectral features.

The L-edge absorption spectrum of Cr(CO)_6_ in Figure 4a is split into the L_3_- and L_2_-edges due to spin–orbit coupling in the core-excited-state final states. The L_3_-edge is characterized by two absorption resonances. Based on our calculations, the less intense pre-edge resonance at 576.5 eV can be assigned to excitations of Cr 2p electrons into unoccupied CO π* orbitals of t_2g_ symmetry. The transitions underlying this resonance carry oscillator strength due to π-backdonation and the associated hybridization of the CO π*(t_2g_) orbitals with the occupied d-derived orbitals of the same symmetry. The stronger main resonance at 578.5 eV is instead due to excitations of Cr 2p electrons into unoccupied 3d-derived orbitals of e_g_ symmetry. Notably, this ordering of resonances at the metal L-edge is different than in other metal carbonyls of lower symmetry^34,73,74^ as well as the isoelectronic ferrous hexacyanide.^75,76^ In these complexes, excitations into the unoccupied d levels are at lower energy than excitations into ligand π* orbitals. This different ordering reflects the higher coordination number in Cr(CO)_6_ compared to other metal carbonyls as well as, compared to ferrous hexacyanide, the carbonyl group being a stronger π acceptor than the negatively charged cyanide group. Overall, this causes a higher ligand field in Cr(CO)_6_ and a destabilization of the unoccupied metal d orbitals to higher energies than the ligand π* orbitals.

The O K-edge spectrum shown in Figure 4b is dominated by a strong absorption resonance at 533.3 eV. Based on our TD-DFT calculations, this resonance can be assigned to excitations of O 1s electrons into the unoccupied CO π* manifold involving specifically orbitals of t_1u_ and t_2u_ symmetry in agreement with previous studies on solution and gas-phase Cr(CO)_6_.^72,77,78^ The low-intensity prepeak at a photon energy of 530.5 eV is instead tentatively assigned to impurities stemming from the sample preparation. Specifically, based on comparison to previous gas-phase electron energy loss spectroscopy measurements,^77,79,80^ the prepeak is most likely due to molecular oxygen dissolved in the octane solution, which was accumulating during the sample preparation (see Methods section).

**Figure 5:**
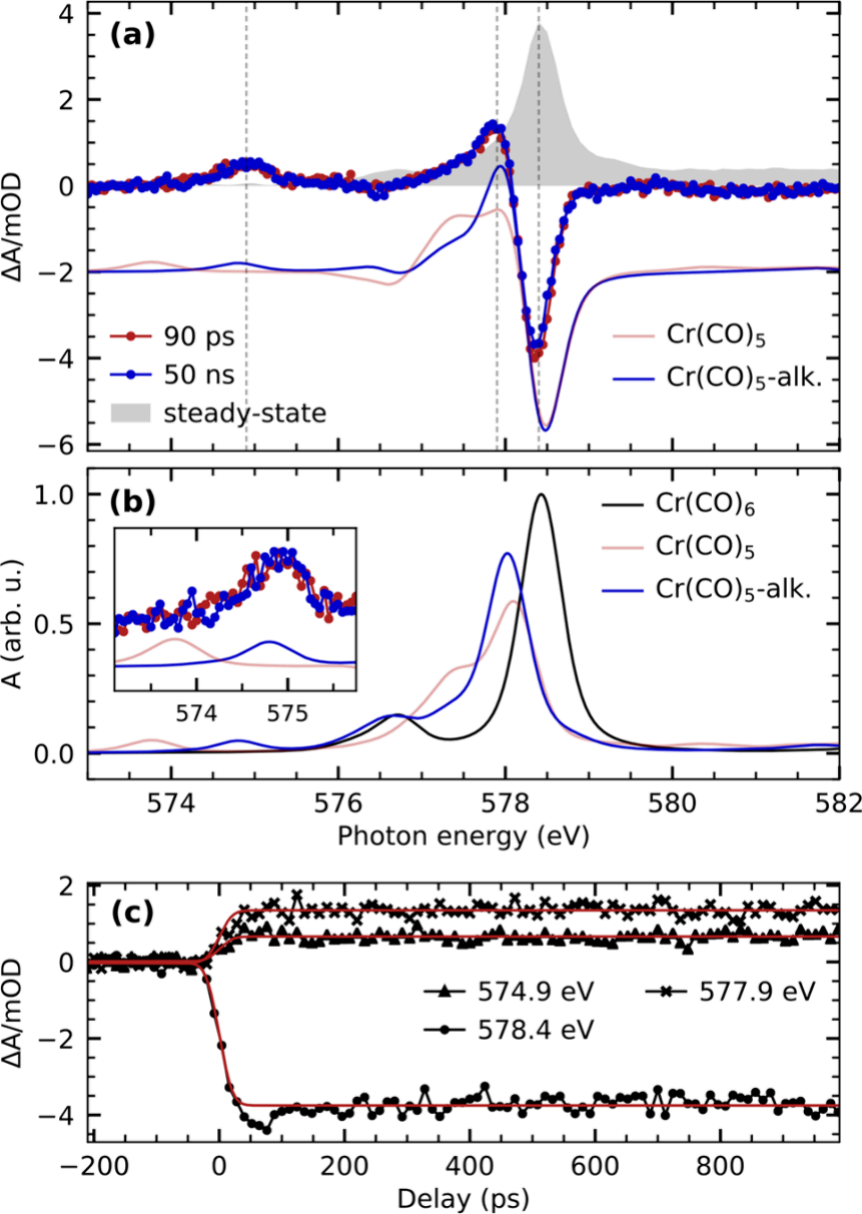
(a) Experimental and calculated transient Cr L_3_-edge absorption difference spectra of Cr(CO)_6_ in octane solution. Calculations are performed at the RASSCF level of theory. Difference spectra of the Cr(CO)_5_-alkane σ-complex and the Cr(CO)_5_ fragment are generated with respect to Cr(CO)_6_ (spectrum of the respective species minus spectrum of Cr(CO)_6_). For better comparison, the depletion of the calculated Cr(CO)_5_-alkane difference spectra is scaled to match the depletion of the experimental spectrum, and the Cr(CO)_5_ difference spectrum is scaled accordingly. The experimental steady-state spectrum is additionally shown for comparison and scaled to match the amplitude of the depletion of the transient difference spectra. (b) Calculated L_3_-edge absorption spectrum of Cr(CO)_6_, the Cr(CO)_5_-alkane σ-complex, and the Cr(CO)_5_ fragment. The maximum of the Cr(CO)_6_ spectrum is scaled to 1, and the other spectra are scaled accordingly. The inset shows a close-up of the transient pre-edge region between 573.1 and 575.75 eV. (c) Pump–probe delay traces measured at the energies indicated in (a). The delay traces are modeled with a step function broadened by a Gaussian function.

Figure 5a shows the transient Cr L_3_-edge absorption spectra of Cr(CO)_6_ in octane solution compared with calculated transient spectra as well as with the Cr(CO)_6_ steady-state spectrum (for comparison, the absolute calculated spectra from which the differences in Figure 5a are generated are shown in Figure 5b). The two experimental difference spectra in Figure 5a were recorded at 90 ps and 50 ns after UV excitation and are identical in shape and intensity. Both spectra exhibit substantial depletion at the energy of the main resonance at 578.4 eV as well as positive transient intensities at 577.9 and 574.9 eV. The delay traces taken at these energies, shown in Figure 5c, are well-described by a step function convolved with a Gaussian function with ∼45 ps fwhm representing the temporal resolution in the X-ray experiments. This is consistent with our transient optical absorption measurements in that the Cr(CO)_5_-alkane σ-complex is expected to form within the time resolution of the X-ray absorption measurements. Both transient X-ray absorption difference spectra thus constitute the electronic-structure fingerprint of the Cr(CO)_5_-alkane σ-complex.

This is again consistent with our calculated Cr L_3_-edge difference spectra of bare Cr(CO)_5_ and the Cr(CO)_5_-alkane σ-complex in Figure 5a. While both calculated difference spectra reproduce the experimentally observed depletion at 578.4 eV and the positive intensity around 577.9 eV, only one of the calculated spectra reproduces the energy of the transient pre-edge peak seen in experiment at 574.9 eV (see inset in Figure 5b). The peak at 574.9 eV is well reproduced by the calculated spectrum of the Cr(CO)_5_-alkane σ-complex, whereas the bare Cr(CO)_5_ fragment has a peak at ∼1 eV lower incidence photon energy. The calculated spectrum of the Cr(CO)_5_-alkane σ-complex additionally shows better agreement in terms of spectral shape around 577.5 eV.

**Figure 6:**
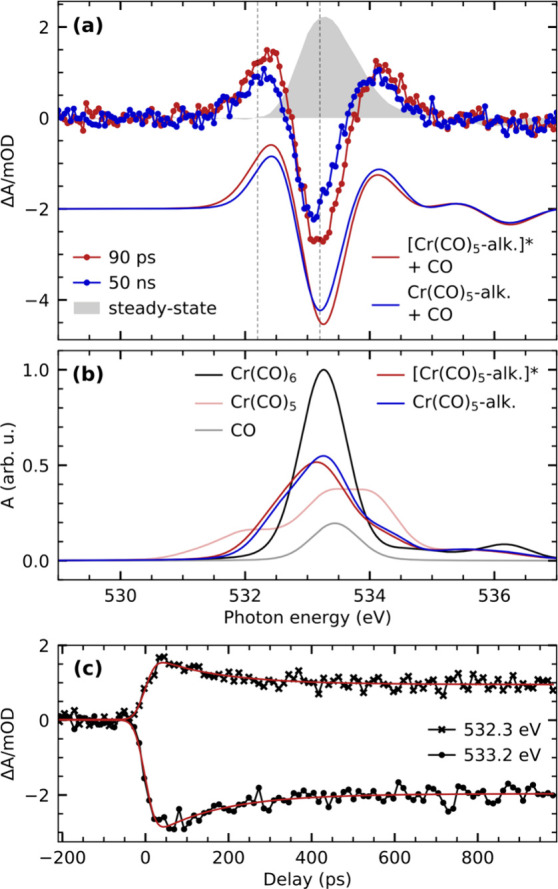
(a) Transient O K-edge absorption difference spectra of Cr(CO)_6_ in octane solution compared with calculated difference spectra of the Cr(CO)_5_-alkane σ-complex and the free CO as well as the Cr(CO)_5_ fragment and the free CO with respect to the Cr(CO)_6_ species. The depletion of the calculated difference spectrum, which represents the sum of the Cr(CO)_5_-alkane σ-complex and the free CO, is scaled to match the depletion of the experimental spectrum at 50 ns. The calculated difference spectrum of the “hot” [Cr(CO)_5_-alkane]* σ-complex plus the free CO is scaled accordingly. The generation of the “hot” [Cr(CO)_5_-alkane]* σ-complex spectrum is discussed in the main text. The experimental steady-state spectrum is additionally shown for comparison and scaled to match the amplitude of the depletion of the transient difference spectrum at 50 ns. (b) Calculated O K-edge absorption spectra of all species considered in generating the difference spectra displayed in (a). The maximum of the Cr(CO)_6_ spectrum is scaled to 1. All other spectra are scaled accordingly. (c) Pump–probe delay traces measured at the energies indicated in (a). The delay traces are modeled with a single-exponential decay of a primary species to a stable product.

Measured O K-edge steady-state and transient difference spectra of Cr(CO)_6_ in octane solution are compared with calculated difference spectra in Figure 6a (absolute calculated spectra are shown in Figure 6b). The two experimental difference spectra (recorded at the same pump–probe delays as at the Cr L_3_-edge) exhibit substantial depletion of the main steady-state absorption resonance at 533.2 eV as well as induced absorption in the pre- and postedge region at 532.3 and 534.2 eV. In contrast to the Cr L_3_-edge difference spectra, however, the amplitudes of the transient difference spectra change as a function of pump–probe delay. The delay traces measured at the transient pre-edge at 532.2 eV as well as the depletion at 533.2 eV are displayed in Figure 6c. Their temporal evolutions are well-described by single-exponential decays with a common time constant of 160 ± 20 ps of a primary species to a secondary metastable species.

The observed signatures are similar to transient O K-edge signatures measured for the photochemistry of Cr(CO)_6_ in 1-pentanol solution. There, the observed absorption differences were assigned to the migration dynamics of C–H to O–H coordination toward the Cr(CO)_5_ species.^72^ In the absence of any functional groups besides C–H bonds in the alkane solution, the here-observed signal changes are instead assigned to the decay of high-frequency CO stretching modes of the σ-complex. These modes have previously been shown to be excited following ligand exchange in Cr(CO)_6_ in alkane solutions.^25,64,81^ The vibrational excitations have been reported to only weakly couple to lower-energy vibrations and to relax on a time scale of 160 ps, in excellent agreement with our measurements.

The comparison of the experimental and theoretical difference spectra in Figure 6a explains how these vibrational excitations are reflected in the O K-edge absorption spectra. The spectrum at 50 ns corresponds to the Cr(CO)_5_-alkane σ-complex plus the dissociated CO ligand. At this delay time, the complex can be safely assumed to be vibrationally cold. At 90 ps instead, the agreement of the calculated spectrum of a “hot” Cr(CO)_5_-alkane σ-complex with experiment at the pre-edge at 532.3 eV as well as at the main-edge at 533.2 eV suggests that the σ-complex is still vibrationally excited. To reflect vibrational excitations in the calculated spectrum, we empirically added a red-shift of 0.1 eV to the incident photon energy of the cold Cr(CO)_5_-alkane σ-complex spectrum. The rationale is that excitation of the CO stretching mode leads to a higher average CO bond length, which stabilizes the CO π* orbitals and shifts O 1s → CO π* resonances to lower energies. We also added an additional 0.1 eV Gaussian broadening to the “hot” Cr(CO)_5_-alkane σ-complex spectrum (in addition to the broadening applied to the spectra of vibrationally cold species, see Computational Details) to reflect the higher degree of conformational motion as a result of the vibrational excitation.^82^

### Probing the Orbital Correlation Diagram

**Scheme 4:**
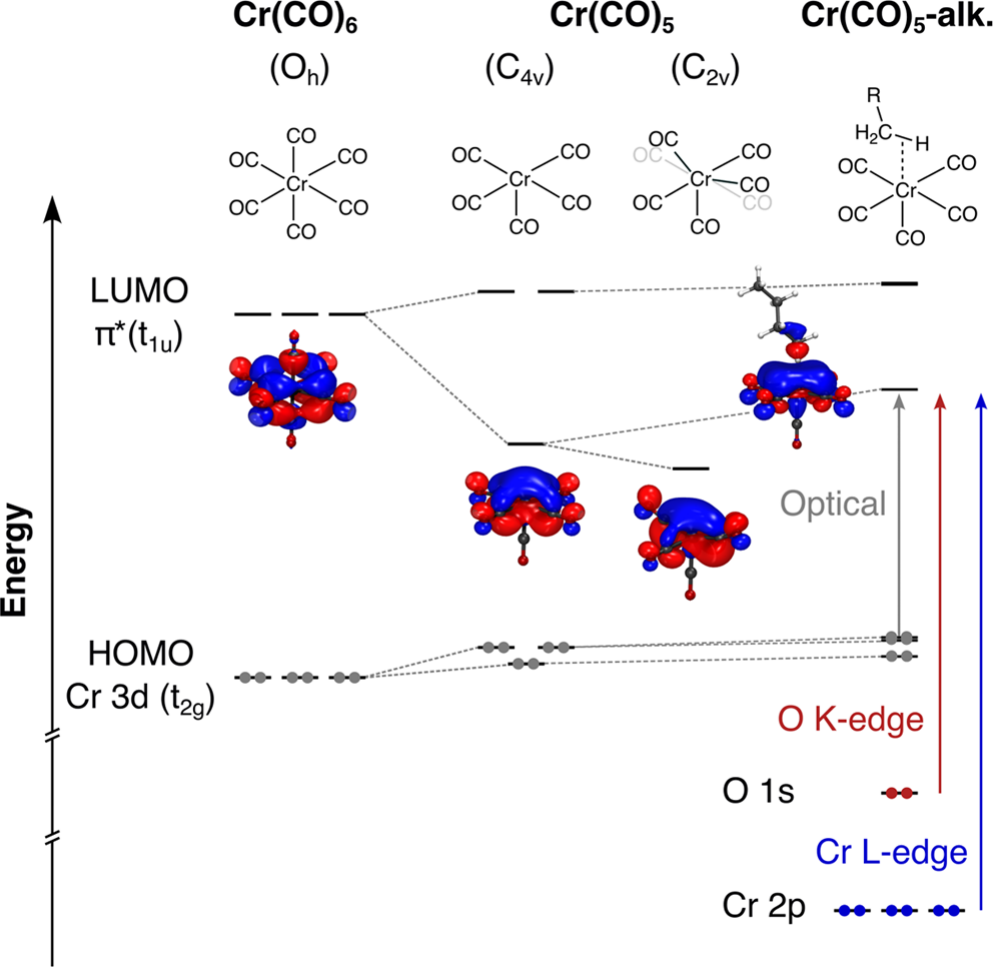
Orbital Correlation Diagram along CO Dissociation from Cr(CO)_6_ to Cr(CO)_5_ and σ-Complex Formation to Cr(CO)_5_-Alkane with Main Optical (Gray), O K-Edge (Red), and Cr L-Edge Absorption Transitions (Blue)

The combination of time-resolved optical and X-ray absorption spectroscopy at the Cr L-edge and O K-edge allows following the evolution of unoccupied valence orbitals upon CO dissociation and alkane σ-complex formation from three different perspectives. A schematic summary of the evolution of the energies of the decisive valence orbitals and how they are accessed by the three different absorption spectroscopy methods is shown in Scheme 4 (a comprehensive orbital correlation diagram of all valence orbitals included in the active space of the RASSCF calculations is shown in Figure S2 in the Supporting Information).

CO dissociation from Cr(CO)_6_ is initially triggered by a HOMO → LUMO metal-to-ligand charge transfer (MLCT) excitation involving Cr 3d (t_2g_) → CO π* (t_1u_) transitions with an absorption band centered at around 290 nm (see Figure 3). CO dissociation breaks the *O*_*h*_ symmetry and is predominantly reflected in a major decrease of energy of one of the former CO π* (t_1u_) orbitals in the Cr(CO)_5_ fragment (see Scheme 4). Since the HOMOs remain relatively stable, the LUMO energy drop can be experimentally observed in the time-resolved optical absorption data (see Figure 3). The structural changes associated with the coherent oscillation of the Cr-CO bending mode further decrease the energy of the LUMO as our calculations suggest and in agreement with experiment (see Scheme 4). Distortion from *C*_4*v*_ to *C*_2*v*_ moves two equatorial CO groups out of plane, which reduces hybridization of the nominal CO π* orbital with the Cr d_*z*^2^_ orbital (see orbital plots in Scheme 4).^68^ This change in orbital overlap stabilizes the LUMO and causes the observed red-shift of the HOMO → LUMO absorption band in *C*_2*v*_ distorted structures (Figure 3). In the Cr(CO)_5_-alkane σ-complex, the bonding interactions between the Cr and the C–H bond increase the LUMO energy, as observed in the blue-shift of the HOMO → LUMO absorption band back to higher energies in the optical absorption spectrum (see Scheme 4 and Figure 3). The degree to which the band shifts thus serves as an experimental measure of the strength of the metal–alkane bonding interaction and is mirrored in the time-resolved X-ray absorption spectra.

Following CO dissociation and alkane association, the LUMO remains an orbital of predominantly CO π* character. It does, however, also adopt some degree of Cr d_*z*^2^_ character due to the discussed changes in Cr-CO hybridization. This emergence of Cr d_*z*^2^_ character is seen in the increase of orbital amplitude of the LUMO at the Cr center when going from Cr(CO)_6_ to the two Cr(CO)_5_ geometries and the Cr(CO)_5_-alkane σ-complex (Scheme 4). By d_*z*^2^_ admixture, the LUMO becomes accessible in Cr L-edge absorption via dipole-allowed 2p → 3d transitions, which can be experimentally observed via the pre-edge peak at 574.9 eV in the transient L_3_-edge spectrum of the Cr(CO)_5_-alkane σ-complex (Figure 5a). Notably, while the transient Cr(CO)_5_ fragment cannot be observed with picosecond X-ray absorption spectroscopy, the calculated shift of the Cr L-edge pre-edge peak of ∼1 eV in the Cr(CO)_5_-alkane σ-complex to higher energy with respect to bare Cr(CO)_5_ mirrors the behavior of the optical absorption. In terms of X-ray versus optical absorption, this shift provides a complementary measure of the strength of the metal–alkane bond.

In terms of the ligand versus metal perspective, the predominant ligand character of the LUMO can be accessed with O K-edge absorption spectroscopy in yet another complementary way. In contrast to the Cr L_3_-edge, the O K-edge probes the LUMO through dipole-allowed transitions of O 1s electrons into the CO π* orbitals, which have substantial O 2p character. The LUMO of the Cr(CO)_5_-alkane σ-complex is observed as a transient pre-edge transition at 532.3 eV (Figure 6a). As in the Cr L_3_-edge, a shift of the pre-edge between the Cr(CO)_5_-alkane σ-complex and the bare Cr(CO)_5_ fragment can be seen (Figure 6b), again reflecting the relative shift in LUMO energy as a result of alkane binding in the Cr(CO)_5_-alkane σ-complex (Scheme 4).

Besides providing complementary insights into the evolution of the LUMO along the photochemical pathway, the time-resolved X-ray absorption data at the Cr L_3_-edge and O K-edge further inform on the evolution of the full manifold of unoccupied Cr 3d and ligand CO π* orbitals, respectively (see Supporting Information for full orbital correlation diagram). The absence of depletion at 576.7 eV in the measured Cr(CO)_5_-alkane σ-complex spectrum, a region of 2p→ CO π*(t_2g_) transitions (Figure 5a), points to the low impact the ligand exchange has on the t_2g_ manifold. Both the Cr(CO)_5_-alkane σ-complex and Cr(CO)_6_ exhibit a similarly intense resonance at the same energy (see Figure 5b), reflecting the unchanged energies of the CO π*(t_2g_) orbitals as well as their unchanged hybridization with Cr 3d orbitals in both species. The Cr L_3_-edge spectrum of the Cr(CO)_5_-alkane σ-complex further reflects the splitting of the 3d(e_g_) orbitals as a result of the breaking of *O*_*h*_ symmetry upon ligand exchange. This can be seen by the splitting of the main-edge resonance in the calculated Cr(CO)_5_-alkane σ-complex spectrum, which, in addition to the main maximum at 578 eV, exhibits a shoulder at 577.2 eV (see Figure 5b). In experiment, this splitting is visible by the extension of the positive transient intensity in Figure 5a around 577.3 eV in addition to the peak centered at 577.8 eV. Intensity in this range is attributed to excitations of Cr 2p electrons into predominantly the d_z^2^_ orbital, whereas the main maximum reflects excitations predominantly into the d_*x*^2^–*y*^2^_ orbital. Both sets of transitions carry less oscillator strength compared to the main resonance in Cr(CO)_6_. This difference is due to the hybridization of CO π* orbitals with the d_*z*^2^_ and d_*x*^2^–*y*^2^_ orbitals in the Cr(CO)_5_-alkane σ-complex, mixing that is allowed in the broken symmetry of Cr(CO)_5_-alkane and forbidden in the *O*_*h*_ symmetry of Cr(CO)_6_. This effect of symmetry breaking is mirrored in the t_2u_ manifold of the CO π* orbitals. Two t_2u_ orbitals of Cr(CO)_6_ are destabilized following ligand exchange (Scheme 4), explaining the induced absorption at 534.2 eV in the O K-edge spectrum of the Cr(CO)_5_-alkane σ-complex (Figure 6a and b).

## Conclusion

We have used a combination of femtosecond optical absorption spectroscopy and picosecond X-ray absorption spectroscopy at the metal L-edge and ligand K-edge to probe the photochemistry and electronic structure evolution of Cr(CO)_6_ in octane solution. The femtosecond optical absorption data allowed unequivocally establishing the photochemical pathway of alkane σ-complex formation from Cr(CO)_6_. Bare Cr(CO)_5_ was found to form within the time resolution of the experiment followed either by geminate recombination with a time constant of 150 fs or octane association to form the Cr(CO)_5_-alkane σ-complex with a time constant of 8.2 ps. Preceding σ-complex formation, the Cr(CO)_5_ fragments, which do not recombine, exhibit coherent oscillations in the optical absorption data. Their frequency and dephasing time are characteristic of equatorial Cr-CO bending modes in the ground state of Cr(CO)_5_, as shown in comparison to the CO dissociation dynamics of gas-phase Cr(CO)_6_. With the robustly established photochemical pathways based on the optical absorption data, we then used X-ray absorption spectroscopy to characterize the electronic structure of the Cr(CO)_5_-alkane σ-complex. The ligand exchange breaks the octahedral symmetry, leading to a rehybridization between metal and ligand orbitals.

We show that substantial shifts of the LUMO energy correlate with several complementary experimental observables and characteristically reflect formation of the undercoordinated metal complex, its structural relaxation to the electronic ground state, and alkane association in a σ-complex. Specifically, the shift in LUMO energy is identified as a novel experiment-based descriptor of the impact of metal–alkane bond formation on the electronic structure in the undercoordinated metal complex. Both time-resolved optical and X-ray absorption spectroscopy can thus be generally established as sensitive probes of the strength associated with the metal–alkane interactions in σ-complexes. This approach can now generally be applied to other σ-complexes to provide a detailed understanding of how varying the stability of metal–alkane bonds in σ-complexes—by choice of metal element and ligand structure—modulates reactivity of σ-complexes. We anticipate future studies, using the orbital-based descriptor of metal–alkane bonding derived here, for precise determination of the mechanistic roles of σ-complexes and their precursors in C–H activation reactions.

## Supporting Information Available

Description of the global fit model; comparison of the residuals of the delay traces at 390 and 670 nm; extended discussion of the origin of the oscillatory signal; details on the line shape model; DFT orbital energies; Cartesian coordinates of the DFT-optimized structures; example input files for the DFT and RASSCF calculations (PDF)
