# Supplementary material for: Photochemical Formation and Electronic Structure of an Alkane σ-Complex from Time-Resolved Optical and X-ray Absorption Spectroscopy
Source: J Am Chem Soc. 2024 May 7;146(20):14000–11. doi: 10.1021/jacs.4c02077 (PMC11117182; doi:10.1021/jacs.4c02077)
Supplement: Supplementary file 1 — ja4c02077_si_001.pdf [file ja4c02077_si_001.pdf]

Supporting Information: Photochemical Formation and Electronic Structure of an Alkane σ-Complex from Time-Resolved Optical and X-ray Absorption Spectroscopy

Raphael M. Jay,* Michael R. Coates, Huan Zhao, Marc-Oliver Winghart, Peng Han, Ru-Pan Wang, Jessica Harich, Ambar Banerjee, Hampus Wikmark, Mattis Fondell, Erik T. J. Nibbering, Michael Odelius,* Nils Huse,* and Philippe Wernet*

Corresponding Author

*Email: raphael.jay@physics.uu.se, odelius@fysik.su.se, nils.huse@uni-hamburg.de, philippe.wernet@physics.uu.se

Description of the global fit model

The UV pump-induced dynamics of $Cr(CO)_6$ are described by an initial fast exponential decay A as well as a slower exponential decay B and exponential rise C (where B and C share the same time constant). All three components are assumed to contribute to a varying degree to the three traces measured at 390 nm, 510 nm and 670 nm. A superposition of contributions from the three components is therefore used to model the individual traces. The individual scaling of the three contributions is determined via additional free fit parameters:

$$\boldsymbol{I_{390\,nm}} = \boldsymbol{a_1}[\boldsymbol{A}] + \boldsymbol{b_1}[\boldsymbol{B}] + \boldsymbol{c_1}[\boldsymbol{C}]$$
$$\boldsymbol{I_{510\,nm}} = \boldsymbol{a_2}[\boldsymbol{A}] + \boldsymbol{b_2}[\boldsymbol{B}] + \boldsymbol{c_2}[\boldsymbol{C}]$$
$$\boldsymbol{I_{670\,nm}} = \boldsymbol{a_3}[\boldsymbol{A}] + \boldsymbol{b_3}[\boldsymbol{B}] + \boldsymbol{c_3}[\boldsymbol{C}]$$

To additionally model the temporal broadening and time-zero of the experiment, the three components are convolved with an error-function broadened by a Gaussian function.

Comparison of residuals

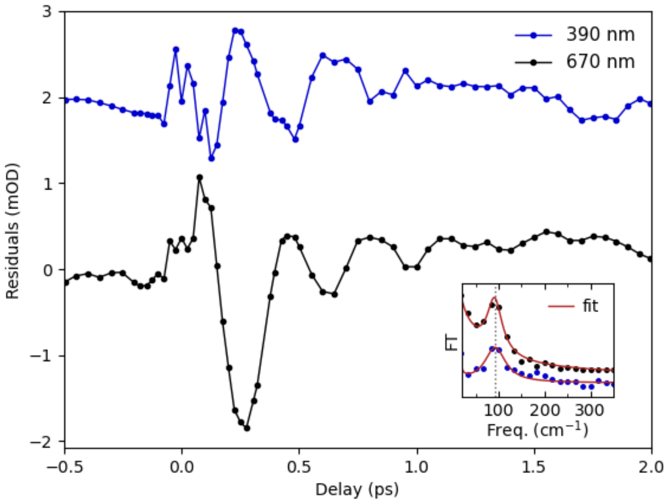

Figure S1 Comparison of the residuals of the global fit at 390 nm and 670 nm. Both residuals show the same oscillatory pattern with a frequency of 91 cm^{-1} indicating that the transient absorption band peaking at wavelengths below 390 nm is a signature of bare $Cr(CO)_5$.

Extended discussion of the origin of the oscillatory signal

The oscillatory features observed in the transient absorption can be attributed to a nuclear wavepacket generated by either (i) impulsive excitation due to vibronic coupling of nuclear degrees of freedom to higher-frequency transitions (e.g. an electronic excitation) or a (ii) displacive excitation, where an excited state is populated in a non-equilibrium geometry from which nuclear dynamics ensue. For the case of UV excitation of $Cr(CO)_6$, four scenarios come to mind:

1. Impulsive excitation creates a Raman wavepacket in the electronic ground-state of the parent molecule $Cr(CO)_6$.
2. Impulsive or displacive excitation creates a Raman wavepacket in the electronically excited state of $Cr(CO)_6$.
3. Displacive excitation creates a wavepacket in an excited state of the product $Cr(CO)_5$.
4. Displacive excitation creates a wavepacket in the ground-state of $Cr(CO)_5$.

Scenario 1 usually modulates the absorption of the parent molecule albeit, also the refractive index of a solution can be modulated. This is particularly true for impulsive Raman excitations of solvent molecules which exist in high concentration and are usually not

in resonance with the pump pulse. Because the modulation clearly modulates the product absorption where the parent molecule $Cr(CO)_6$ does not absorb (see Fig. 3a) we do not attribute this modulation to the parent molecule $Cr(CO)_6$ in its electronic ground state.

Scenario 2 can also be ruled out in our view because previous studies in gas-phase show that the antibonding state, which leads to CO dissociation, is populated in tens of femtoseconds according to Trushin et al[1,2]. Hence, the product forms in less than 100 fs.

Scenario 3 could indeed occur since the ballistic wavepacket dynamics lead to CO dissociation and population of the excited 11E state of $Cr(CO)_5$, according to Burdet et al[3]. However, this state is strongly coupled to lower-lying states due to state degeneracy such that the Jahn-Teller effect permits electronic relaxation to the ground-state of $Cr(CO)_5$ within ~100 fs after initial excitation if we follow the time intervals of ballistic motion in Trushin et al[2].

Scenario 4 is therefore the scenario that we think causes the sinusoidal modulation of the induced absorption in the residuals of Fig. S1. We also note that the extracted frequency of (91 ± 1) cm^{-1} disagrees with a measured Raman transition of $Cr(CO)_6$ in solution reported by Jones et al[4] but is in better agreement with Trushin et al[2], where the authors also rationalize why scenario 4 seems the most likely one.

Details on the line shape model

To model the line shape of the transient absorption band emerging in the visible range of the absorption spectrum, an asymmetric Gaussian line shape with a width w_0 at the position x_0 on top of a 1/x-shaped background was used, which is described by the following fit function:

$$f(x) = \frac{A}{\sqrt{2\pi} \cdot w} \cdot e^{\frac{-(x-x_0)^2}{2w^2}} + \frac{c}{x-d}$$

With

$$w = \frac{w_0}{(1 + e^{a(x-x_0)}) \cdot \sqrt{2ln2}}$$

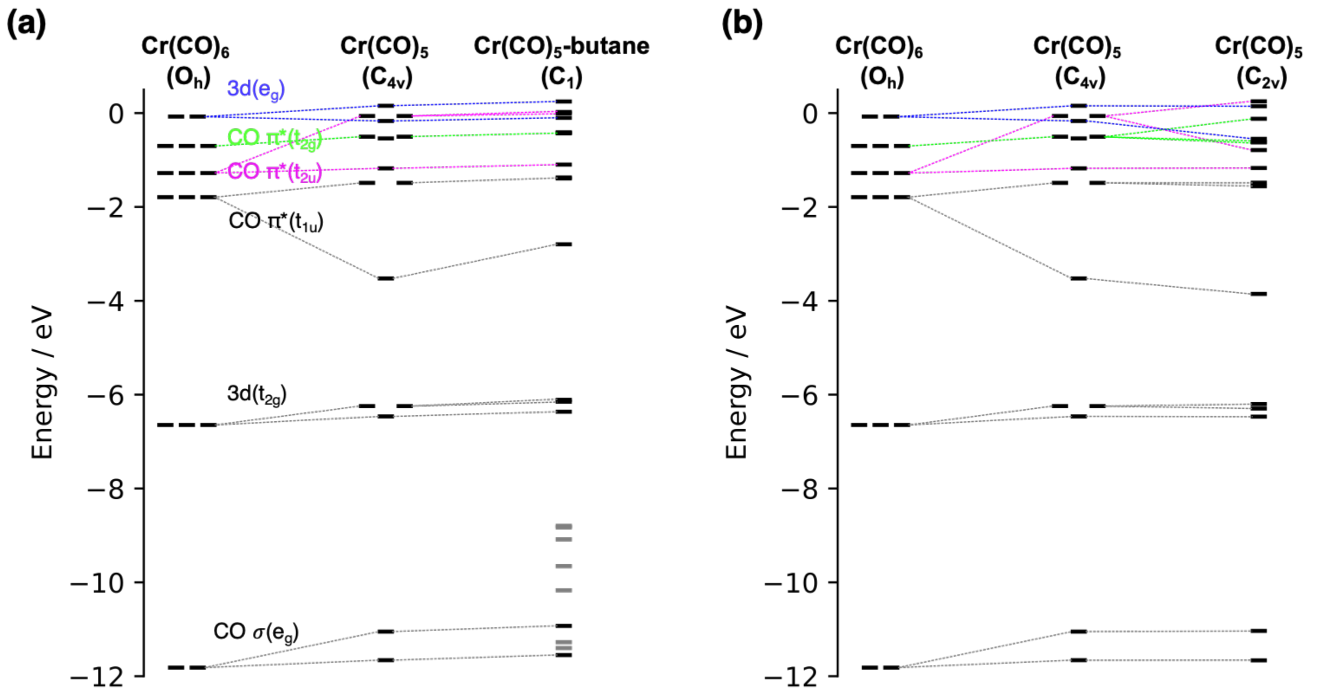

Figure S2. Orbital energies from the optimized structures at the DFT TPSSh level of theory of the orbitals included in the restricted active space of the RASSCF calculations. **(a)** Orbitals of $Cr(CO)_6$ compared with $Cr(CO)_5$ in C_{4v} symmetry as well as the $Cr(CO)_5$-butane σ-complex. **(b)** Orbitals of $Cr(CO)_6$ compared with $Cr(CO)_5$ in C_{4v} as well as C_{2v} symmetry.

Optimized molecular geometries (coordinates given in Å)

$Cr(CO)_6$ (O_h)

Cr	0.000000	-0.000000	-0.000000
C	-0.000000	0.000000	1.910608
C	0.000000	-0.000000	-1.910608
C	-0.000000	1.910608	-0.000000
C	0.000000	-1.910608	0.000000
C	1.910608	0.000000	0.000000
C	-1.910608	-0.000000	-0.000000
O	-0.000000	0.000000	3.054682
O	0.000000	-0.000000	-3.054682
O	-0.000000	3.054682	-0.000000
O	0.000000	-3.054682	0.000000
O	3.054682	0.000000	0.000000
O	-3.054682	-0.000000	-0.000000

$Cr(CO)_5$ (C_{4v})

Cr	0.0000000	0.0000000	-0.0304845
C	0.0000000	0.0000000	1.8038421
C	0.0000000	1.9124424	-0.0133066
C	0.0000000	-1.9124424	-0.0133066
C	1.9124424	0.0000000	-0.0133066
C	-1.9124424	0.0000000	-0.0133066
O	0.0000000	0.0000000	2.9578440
O	0.0000000	3.0558276	0.0641629
O	0.0000000	-3.0558276	0.0641629
O	3.0558276	0.0000000	0.0641629
O	-3.0558276	0.0000000	0.0641629

$Cr(CO)_5$ (C_{2v})

Cr	0.0000000	0.0000000	0.1918006
C	0.0000000	0.0000000	2.2706429
C	0.0000000	1.3242298	-1.0820054
C	0.0000000	-1.3242298	-1.0820054
C	1.8943904	0.0000000	0.0338687
C	-1.8943904	0.0000000	0.0338687
O	0.0000000	0.0000000	3.4024997
O	0.0000000	2.1828040	-1.8540423
O	0.0000000	-2.1828040	-1.8540423
O	3.0263447	0.0000000	-0.1562280
O	-3.0263447	0.0000000	-0.1562280

$Cr(CO)_5$ (C_{2v} distortion)

Cr	0.0000000	0.0000000	-0.0524864
C	0.0000000	0.0000000	1.7874789
C	0.0000000	1.8652263	-0.5216482
C	0.0000000	-1.8652263	-0.5216482
C	1.8990009	0.0000000	0.0865771
C	-1.8990009	0.0000000	0.0865771
O	0.0000000	0.0000000	2.9405475
O	0.0000000	2.9172899	-0.9728457
O	0.0000000	-2.9172899	-0.9728457
O	3.0328477	0.0000000	0.2610441
O	-3.0328477	0.0000000	0.2610441

$Cr(CO)_5$-butane

Cr	-3.6047810016	0.422232927	-2.4999834401
C	-4.2919246903	-0.6368588604	-3.9331096034
C	-1.8557837776	-0.2810484034	-2.8038708877
C	-2.9128855425	1.5170260029	-1.0994209204
C	-5.3437717624	1.1560349856	-2.2207755464
C	-3.2501247316	1.780513902	-3.6940208761
O	-4.6961060556	-1.2295085145	-4.8274197768
O	-0.795134922	-0.6593041405	-3.020322709
O	-2.4865739039	2.2175734758	-0.2977068267
O	-6.3765375161	1.6364548784	-2.0888620604
O	-3.0302571253	2.6339008834	-4.4380273576
H	-2.6938615611	-5.1037131503	2.1220433091
C	-2.6877160542	-4.0216422428	1.9700586682
H	-3.1919538787	-3.5600642948	2.8239659105
H	-1.6461913012	-3.6876358006	1.9771180997
C	-3.3753337205	-3.6452499841	0.6579700539
H	-2.8710351127	-4.1433526495	-0.177928877
H	-4.406378501	-4.016752384	0.6635504262
C	-3.3855616106	-2.1347186549	0.4108410556
H	-3.8901604677	-1.6316801823	1.2425013195
H	-2.3567004956	-1.7605810846	0.395487283
C	-4.0802366011	-1.7726459735	-0.9019573134
H	-5.1234112117	-2.0935724015	-0.9206809173
H	-4.1249133067	-0.6518519128	-0.957499897
H	-3.5711141484	-2.2190644208	-1.7551101162

CO

C	0.000000	0.000000	1.916631
O	0.000000	0.000000	3.045822

Example input files for $Cr(CO)_6$

Geometry optimizations

```
%chk=1CrCO6_TPSSH_DEF2-TZVP_Oh.chk
%NProcShared=16
%Mem=80000MB
# TPSSh/def2TZVP OPT FREQ Symm=(loose) scrf(cpcm, solvent=n-Octane)

Text line Below the charge and multiplicity is followed by coordinates in Å

 0  1
 Cr      0.000000     0.000000     0.000000
 C       0.000000     0.000000     1.903886
 C       0.000000     0.000000    -1.903886
 C       0.000000     1.903886     0.000000
 C       0.000000    -1.903886     0.000000
 C       1.903886     0.000000     0.000000
 C      -1.903886     0.000000     0.000000
 O       0.000000     0.000000     3.058567
 O       0.000000     0.000000    -3.058567
 O       0.000000     3.058567     0.000000
 O       0.000000    -3.058567     0.000000
 O       3.058567     0.000000     0.000000
 O      -3.058567     0.000000     0.000000
```

Optical absorption spectra

```
!B3LYP def2-TZVP def2/J TIGHTSCF RIJCOSX

%pal
nprocs 16
end

%tddft
NRoots      30
end

* xyzfile 0 1 CrCO6.xyz
```

O K-edge absorption spectra

```
!B3LYP def2-TZVP def2/J TIGHTSCF RIJCOSX

%pal
nprocs 16
end

%tddft
```

```
NRoots         180
MaxDim          900
OrbWin[0] = 5,10,-1,-1
XASloc[0] = 5,10
DoQuad        True
end

* xyzfile 0 1 CrCO6.xyz
```

Cr L-edge absorption spectra

The calculations were performed by starting with the ANO-RCC-MB basis set, rotating the relevant orbitals and then expanding the basis to the ANO-RCC-VTZP level of theory using the &EXPBAS scheme in MOLCAS.

```
&GATEWAY
 coord = 1CrCO6.xyz
BASIS= ANO-RCC-MB
group=nosymm
ANGM
0. 0. 0.
Douglas-Kroll
AMFI
SDIP
ricd
End of input
*------------------------------------------------------------------------------
&SEWARD &END
End of input
*------------------------------------------------------------------------------
&SCF &END
End of input
*------------------------------------------------------------------------------
&RASSCF &END
Lumorb
alter=4;1 47 50;1 48 51;1 64 67;1 65 68
symmetry=1
Spin=1
nActEl
10 0 2
Inactive
  49
Ras1
   0
Ras2
   5
Ras3
   11
CIONLY
CIRO
1 1 1
```

```
End of input
*-------------------------------------------------------------------------------
&RASSCF &END
Lumorb
symmetry=1
Spin=1
nActEl
10 0 2
Inactive
  49
Ras1
   0
Ras2
   5
Ras3
   11
CIRO
1 1 1
End of input
*-------------------------------------------------------------------------------
&RASSCF &END
LUMORB
symmetry=1
Spin=1
SUPSYM
1
3 3 4 5
ALTER
3
1 3 47
1 4 48
1 5 49
nActEl
16 1 2
Inactive
  46
Ras1
   3
Ras2
   5
Ras3
   11
CIONLY
CIRO
1 1 1
End of input
>> COPY $Project.RasOrb $Project.RasOrb_VE_Spin1.Sym1
>> COPY $Project.JobIph $Project.JobIph_VE_Spin1.Sym1
*-------------------------------------------------------------------------------
&RASSCF &END
SXDAmp= 0.002
```

```
LEVSHFT = 1.5
THRS = 0.1e-03, 0.1e-01 , 0.1e-04
LUMORB
symmetry=1
Spin=1
SUPSYM
1
3 47 48 49
nActEl
16 1 2
Inactive
  46
Ras1
   3
Ras2
   5
Ras3
   11
CIRO
80 80 1
End of input
>> COPY $Project.RasOrb $Project.RasOrb_VE_Spin1.Sym1
>> COPY $Project.JobIph $Project.JobIph_VE_Spin1.Sym1
*-----------------------------------------------------------------------------
>>> COPY $Project.RunFile RUNFIL1
*-----------------------------------------------------------------------------
&GATEWAY
 coord = 1CrCO6.xyz
BASIS= ANO-RCC-VTZP
group=nosymm
ANGM
0. 0. 0.
Douglas-Kroll
AMFI
SDIP
ricd
End of input
>>> COPY $Project.RunFile RUNFIL2
*-----------------------------------------------------------------------------
&EXPBAS
  FileOrb = $Project.RasOrb
*-----------------------------------------------------------------------------
>>> RM RUNFIL1
>>> RM RUNFIL2
*-----------------------------------------------------------------------------
&SEWARD
*-----------------------------------------------------------------------------
&RASSCF &END
Fileorb=$Project.ExpOrb
symmetry=1
Spin=1
```

```
SUPSYM
1
3 47 48 49
nActEl
16 1 2
Inactive
  46
Ras1
   3
Ras2
   5
Ras3
   11
CIRO
80 80 1
End of input
>> COPY $Project.RasOrb $Project.RasOrb_VE_Spin1.Sym1
>> COPY $Project.JobIph $Project.JobIph_VE_Spin1.Sym1
*-----------------------------------------------------------------------------
>> foreach MULTIPLICITY in ( 1, 3 )
>> foreach SYMMETRY in ( 1 )
&RASSCF &END
LUMORB
symmetry=$SYMMETRY
Spin=$MULTIPLICITY
SUPSYM
1
3 47 48 49
nActEl
16 1 2
Inactive
  46
Ras1
   3
Ras2
   5
Ras3
   11
CIRO
80 80 1
End of input
>> COPY $Project.RasOrb $Project.RasOrb_VE_Spin$MULTIPLICITY.Sym$SYMMETRY
>> COPY $Project.JobIph $Project.JobIph_VE_Spin$MULTIPLICITY.Sym$SYMMETRY
>> enddo
>> enddo
*-----------------------------------------------------------------------------
>> foreach MULTIPLICITY in ( 1 )
>> foreach SYMMETRY in ( 1 )
&RASSCF &END
SDAV=241
LUMORB
```

```
symmetry=$SYMMETRY
Spin=$MULTIPLICITY
SUPSYM
1
3 47 48 49
nActEl
16 1 2
Inactive
  46
Ras1
   3
Ras2
   5
Ras3
   11
HEXS; 1; 1;
CIRO
240 240 1
End of input
>> COPY $Project.RasOrb $Project.RasOrb_CE_Spin$MULTIPLICITY.Sym$SYMMETRY
>> COPY $Project.JobIph $Project.JobIph_CE_Spin$MULTIPLICITY.Sym$SYMMETRY
>> enddo
>> enddo
*-----------------------------------------------------------------------------
>> foreach MULTIPLICITY in ( 3 )
>> foreach SYMMETRY in ( 1 )
&RASSCF &END
SDAV=481
LUMORB
symmetry=$SYMMETRY
Spin=$MULTIPLICITY
SUPSYM
1
3 47 48 49
nActEl
16 1 2
Inactive
  46
Ras1
   3
Ras2
   5
Ras3
   11
HEXS; 1; 1;
CIRO
480 480 1
End of input
>> COPY $Project.RasOrb $Project.RasOrb_CE_Spin$MULTIPLICITY.Sym$SYMMETRY
>> COPY $Project.JobIph $Project.JobIph_CE_Spin$MULTIPLICITY.Sym$SYMMETRY
>> enddo
```

```
>> enddo
*------------------------------------------------------------------------------
>> COPY $Project.JobIph_VE_Spin1.Sym1 JOB001
>> COPY $Project.JobIph_VE_Spin3.Sym1 JOB002
>> COPY $Project.JobIph_CE_Spin1.Sym1 JOB003
>> COPY $Project.JobIph_CE_Spin3.Sym1 JOB004
 &RASSI  &END
SpinOrbit
Ejob
SUBS=160
TRDI
TRDC
End of input
*------------------------------------------------------------------------------
```

References

(1) Trushin, S. A.; Fuss, W.; Schmid, W. E.; Kompa, K. L. Femtosecond Dynamics and Vibrational Coherence in Gas-Phase Ultraviolet Photodecomposition of Cr(CO)6. *J. Phys. Chem. A* **1998**, *102* (23), 4129–4137. https://doi.org/10.1021/jp973133o.

(2) Trushin, S. A.; Kosma, K.; Fuß, W.; Schmid, W. E. Wavelength-Independent Ultrafast Dynamics and Coherent Oscillation of a Metal–Carbon Stretch Vibration in Photodissociation of Cr(CO)6 in the Region of 270–345nm. *Chem. Phys.* **2008**, *347* (1–3), 309–323. https://doi.org/10.1016/j.chemphys.2007.09.057.

(3) Burdett, J. K.; Grzybowski, J. M.; Perutz, R. N.; Poliakoff, M.; Turner, J. J.; Turner, R. F. Photolysis and Spectroscopy with Polarized Light: Key to the Photochemistry of Pentacarbonylchromium and Related Species. *Inorg. Chem.* **1978**, *17* (1), 147–154. https://doi.org/10.1021/ic50179a028.

(4) Jones, L. H.; McDowell, R. S.; Goldblatt, M. Force Constants of the Hexacarbonyls of Chromium, Molybdenum, and Tungsten from the Vibrational Spectra of Isotopic Species. *Inorg. Chem.* **1969**, *8* (11), 2349–2363. https://doi.org/10.1021/ic50081a025.
